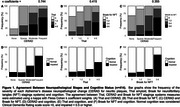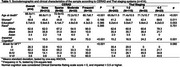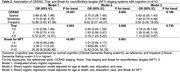# Evaluating Amyloid Pathology and Cognitive Outcomes in AD: Insights from CERAD and Thal Staging

**DOI:** 10.1002/alz70856_100810

**Published:** 2025-12-25

**Authors:** Alberto Fernando Oliveira Justo, Vitor Ribeiro Paes, Renata Elaine Paraizo Leite, Carlos Augusto Pasquallucci, Eduardo Ferriolli, Ricardo Nitrini, Lea T. Grinberg, Claudia Kimie Suemoto

**Affiliations:** ^1^ Physiopathology in Aging Laboratory (LIM‐22), University of Sao Paulo Medical School, São Paulo, São Paulo, Brazil; ^2^ Physiopathology in Aging Laboratory (LIM‐22), Department of Internal Medicine, University of São Paulo Medical School, São Paulo, São Paulo, Brazil; ^3^ University of São Paulo Medical School, São Paulo, São Paulo, Brazil; ^4^ University of Sao Paulo Medical School, São Paulo, Brazil; ^5^ University of São Paulo, São Paulo, Brazil; ^6^ Memory and Aging Center, Weill Institute for Neurosciences, University of California, San Francisco, San Francisco, CA, USA; ^7^ Division of Geriatrics, Department of Internal Medicine, University of São Paulo Medical School, São Paulo, São Paulo, Brazil

## Abstract

**Background:**

The Consortium to Establish a Registry for Alzheimer's Disease (CERAD) and Thal staging are the most used systems to evaluate amyloid pathology for Alzheimer's Disease (AD). This study aims to compare these two systems in assessing amyloid pathology and their correlation with cognitive outcomes and tau pathology, as evaluated through Braak staging for neurofibrillary tangles (Braak NFT).

**Method:**

This is a cross‐sectional population‐based autopsy using samples from the Biobank for Aging Studies collected between 2004 and 2024. Participants did not have significant vascular lesions, Lewy body disease, or Limbic‐predominant age‐related TDP‐43 encephalopathy (LATE). Binary logistic regression models were employed, using CERAD, Thal, or Braak for NFT as exposure variables and clinical dementia rating (CDR; 0 for normal cognition and >0 for impaired cognition) as the outcome variable, adjusted for age at death, sex, education, race, and Braak NFT (when applicable). The kappa coefficient was calculated to analyze the agreement between neuropathological stages.

**Result:**

Participants with higher CERAD and Thal scores were generally older, predominantly women, less educated, and white. Individuals with frequent neuritic plaques exhibited worse cognitive abilities and higher Braak NFT scores (Table 1). Notably, less than 50% of participants with elevated Thal scores showed impaired cognition. CERAD scores were associated with poorer cognition (sparse OR:0.70, 95%CI:0.28–1.55; moderate OR:2.70, 95%CI:1.17–5.98; frequent OR:8.02, 95%CI:2.01–40.37; p for trend=0.002). Conversely, associations with Thal staging were borderline (*p* for trend=0.050). Braak NFT was associated with poorer cognition (stages I‐II OR:1.01, 95%CI:0.49–2.17; III‐IV OR:1.53, 95%CI: 0.59–3.67; V‐VI OR:12.42, 95%CI:3.34–54.18; p for trend=0.001). However, when adjusted for Braak NFT the associations of cognition with CERAD (*p* for trend=0.180) or Thal (*p* for trend=0.730) were no longer significant (Table 2). Agreement analysis revealed kappa coefficients of 0.744 between CERAD and Thal, 0.415 between Thal and Braak, and 0.355 between CERAD and Braak, with higher stages associated with increased cognitive impairment (Figure 1).

**Conclusion:**

CERAD scores were associated with cognition, while Thal scores were not. However, after adjusting for Braak NFT, this association was no longer significant, suggesting that the frequency of amyloid deposits may have a closer correlation with neurofibrillary tangles classification than Thal staging.